# Pilot Study of an Integrated Gait and Spine Kinematics Protocol Using Optoelectronic Motion Analysis in Scoliosis Patients: Validation, Usability, and Comparison with Healthy Controls

**DOI:** 10.3390/bioengineering13040419

**Published:** 2026-04-02

**Authors:** Luca Emanuele Molteni, Luigi Piccinini, Riccardo Riboni, Giuseppe Andreoni

**Affiliations:** 1 Scientific Institute IRCCS “E. Medea”, Bosisio Parini, 23842 Lecco, Italy; 2 Department of Design, Politecnico di Milano, 20158 Milano, Italy

**Keywords:** spine, gait, integrated functional assessment, thoracic spine, lumbar spine, kinematic motion analysis

## Abstract

Background: Gait analysis offers a comprehensive assessment of locomotion and postural control, which are often altered in individuals with spinal deformities. After validating a stereophotogrammetric protocol for whole-body kinematics, including spinal motion in healthy subjects, its application to clinical populations is needed to assess its clinical relevance. Patients treated with spinal arthrodesis for scoliosis may show reduced trunk mobility and compensatory gait strategies. Methods: The validated spinal protocol was applied to 10 patients with scoliosis who underwent arthrodesis and 5 healthy controls. For each participant, the range of motion (ROM) of the upper thoracic, lower thoracic, and lumbar districts was computed. Group differences were assessed with the Mann–Whitney U test, and time-normalized angular curves were compared using Statistical Parametric Mapping (SPM1d). Results: In the pathological group, the protocol showed moderate-to-excellent intra- and inter-operator reliability (ICC > 0.594). Compared with controls, patients exhibited a significant reduction in ROM in fused or adjacent districts. SPM analysis identified altered upper thoracic flexion–extension patterns, particularly relative to the lower thoracic segment, throughout the gait cycle. Conclusions: The protocol demonstrated preliminary feasibility and sensitivity in identifying segmental and phase-dependent changes in spinal motion after arthrodesis, indicating that it may serve as a useful tool for exploratory postoperative gait evaluation.

## 1. Introduction

Quantitative analysis of human movement has progressively become an essential tool in clinical and rehabilitation research. Among the available technologies, marker-based optoelectronic systems are currently considered the gold standard for motion analysis due to their high accuracy, repeatability, and ability to capture dynamic motor tasks at high sampling frequencies [1,2,3]. Through stereophotogrammetry, joint and segmental kinematics can be reconstructed during activities of daily living (ADLs) using a non-invasive laboratory-based approach [4,5,6,7], where reflective markers placed on anatomical landmarks are tracked by infrared camera systems and processed through biomechanical models.

Gait analysis represents the most established application of stereophotogrammetric techniques [2]. Walking is not only a cyclic locomotor task but also the result of complex interactions between neural control, musculoskeletal structures, and sensory feedback mechanisms. Consequently, a comprehensive assessment of gait should not be limited to lower-limb mechanics but should also include a detailed evaluation of trunk and spinal motion, which plays a critical role in postural control and balance.

Nevertheless, many gait analysis protocols currently adopted in clinical practice focus primarily on the lower extremities. Widely used biomechanical models [7,8] typically describe pelvis and lower-limb kinematics, while the trunk, when included, is often simplified as a single rigid segment [9]. This representation neglects the functional role of the spine as a multi-articulated structure, which is fundamental for maintaining balance, enabling trunk mobility during ADLs [10,11], and coordinating head and upper-limb motion.

Interest in multisegmental spinal kinematics has increased over the last decade, with studies investigating trunk motion in static standing [12,13] or during controlled exercises [14,15,16]. However, translating these approaches to gait analysis has proven challenging. Several proposed models either rely on a large number of skin markers, limiting feasibility and repeatability in clinical populations, or focus on isolated spinal regions, such as the thoracic or lumbar spine alone [17,18,19,20,21,22]. Moreover, spinal motion during walking is frequently described in only one or two anatomical planes [23,24,25], despite the three-dimensional nature of trunk control. Only a limited number of studies have modeled the upper thoracic, lower thoracic, and lumbar regions simultaneously in all three planes [26,27], and systematic assessments of measurement reliability during gait remain scarce.

Scoliosis is the most common spinal disorder in children and adolescents, with an overall prevalence of 0.47–5.2% in the current literature [28]. This pathology impacts most of the motor capabilities, including gait. Functional evaluation of scoliosis gait assesses how spinal curvature alters movement, typically revealing asymmetrical trunk rotation, reduced muscle efficiency (23–32% lower), and increased gait energy expenditure. Analysis often uses 3D motion capture to measure kinematic, kinetic, and reaction forces to quantify gait adaptations, such as a stiffer walking pattern [29,30]. Yet, prior analyses generally assess gait without specifically quantifying multisegmental spinal motion in three dimensions, limiting the ability to detect subtle compensatory strategies or post-surgical changes.

To address this gap, a dedicated marker-based protocol for whole-body gait analysis with multisegmental spine representation was recently developed and validated in healthy individuals. Starting from the clinically established Davis marker set [31], a reduced number of additional trunk markers was introduced to characterize spinal kinematics while preserving practicality and clinical usability. That study demonstrated satisfactory accuracy and intra- and inter-operator reliability, supporting the protocol’s methodological robustness [32].

Despite these promising results, the applicability of this protocol in clinical populations with altered spinal mechanics remains poorly investigated. In particular, patients with scoliosis treated by spinal arthrodesis represent a relevant clinical model for studying spinal kinematics during gait. Surgical fusion can restrict segmental spinal mobility and induce compensatory adaptations in adjacent segments as well as in overall trunk motion during walking. Furthermore, to our knowledge, only a few studies have investigated the kinematics of different spinal segments across multiple anatomical planes during gait [5,13,14,21,23,25].

Therefore, the aim of the present study was to apply this integrated gait and spine kinematic protocol to a cohort of post-arthrodesis scoliosis patients in order to evaluate the repeatability and clinical feasibility of the protocol in a pathological population. Moreover, the study aimed to compare the spinal kinematics of post-arthrodesis scoliosis patients with those of healthy controls.

## 2. Materials and Methods

### 2.1. Subjects and Sample Size Determination

Following the prior validation of the spinal kinematics protocol in healthy subjects [32], the present study recruited a clinical sample to evaluate its applicability and reliability in a pathological population. The study involved 1 male and 9 females who had undergone spinal arthrodesis for scoliosis (Table 1). Furthermore, the study involved a small sample of 5 normal subjects, to compare the outcome between the two groups. Patients were recruited from the Scientific Institute E. Medea. Inclusion criteria were the ability to walk independently and the absence of other musculoskeletal or neurological disorders affecting gait. Furthermore, the mean time since surgery was 19.3 ± 4.4 days (range: 11–28 days). In most patients, the spinal fusion involved both thoracic and lumbar segments, reflecting the typical surgical treatment for spinal deformity. Only one patient presented a limited fusion at the L5–S1 level. Although detailed surgical parameters such as pre- and postoperative Cobb angles or instrumentation type were not available for all subjects, the cohort was characterized by relatively comparable post-surgical conditions in terms of fusion extent and recovery time. Controls had no history of spinal pathology or lower limb impairment. All participants provided written informed consent (for minors, consent was signed by parents). The study was approved by the Institutional Ethics Committee (Study ID: 0053, date of approval 18 May 2023).

### 2.2. Data Collection

**Figure 1:**
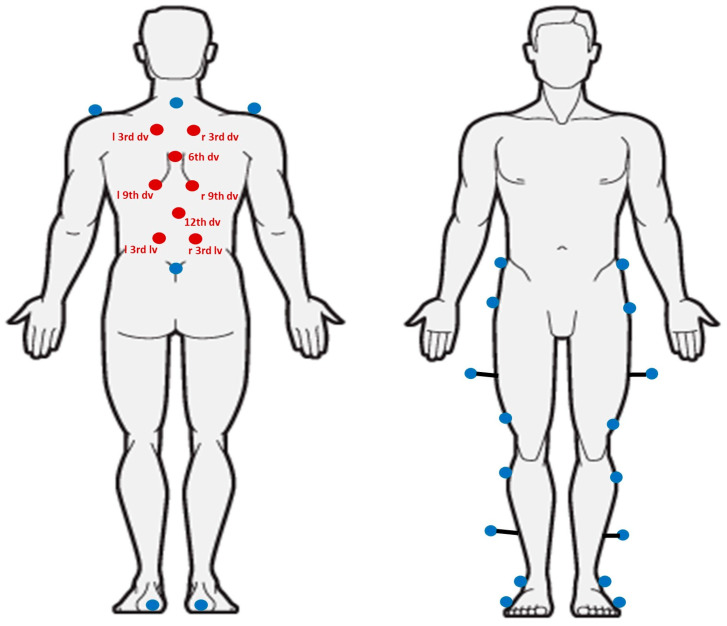
Positions of the markers on the subject. The blue markers represent the markers of the Davis protocol; the red markers represent the markers added to the spine motion analysis (dv: dorsal vertebrae, lv: lumbar vertebrae).

All gait measurements were performed at IRCCS E. Medea using an optoelectronic motion capture system (SMART DX, BTS, Milan, Italy) equipped with eight infrared cameras operating at 100 Hz. A total of 30 reflective markers (plastic spheres with 10 mm diameter coated with reflective film) were placed on each participant: 22 markers according to the Davis protocol [31] and 8 dedicated to spinal motion. The placement of spinal markers followed the configuration described in a previous study conducted in healthy individuals [32] (Figure 1), enabling the assessment of upper thoracic, lower thoracic, and lumbar segments while maintaining compatibility with the standard Davis protocol.

Participants performed five barefoot walking trials along a 6 m walkway at a self-selected comfortable pace. Clinical operators with prior training in spinal landmark identification applied the markers and conducted all acquisitions, ensuring consistency with the validated protocol in healthy subjects [32], enabling comparison between the pathological and control groups. This approach allowed for direct comparison between pathological and control groups and supports the reproducibility of measurements, including both biological replicates (repeated trials for each participant) and technical replicates (marker placement consistency).

For intra- and inter-operator validation, multiple consecutive sessions were conducted for each subject, with approximately a five-minute break between sessions. Each session followed the same protocol:

- Preparation of the subject by the first operator, including the placement of 30 reflective markers on the selected body landmarks and the collection of anthropometric measurements.
- Acquisition of 5 walking trials, in which the participant walked 6 m at a self-selected comfortable pace.
- Removal of the markers from the subject.

In total, each participant completed 2 sessions with 2 different operators, resulting in 20 walking trials per subject for validation purposes. The 20 walking trials collected across the four sessions were used to assess intra- and inter-operator reliability. For the comparison between healthy and post-arthrodesis scoliosis patients, only the first session of each subject was used to ensure consistency.

### 2.3. Data Processing

Raw marker trajectories were processed using Smart Analyzer software 1.10.0470 (BTS Bioengineering, Milan, Italy). Short gaps in marker tracking were interpolated, and data were filtered to reduce noise.

Spinal kinematics were computed for three main segments: upper thoracic, lower thoracic, and lumbar, using a segmental model based on Euler angles in the sagittal (flexion–extension), frontal (lateral bending), and transverse (axial rotation) planes, consistent with the International Society of Biomechanics’ recommendations [33]. For each segment, the following parameters were calculated:

- Sagittal Vertical Axis (SVA) was defined as the horizontal distance between the S1 marker and a vertical line dropped from the C7 marker in the sagittal plane.
- Coronal Vertical Axis (CVA) was defined as the horizontal distance between the S1 marker and a vertical line dropped from the C7 marker in the coronal plane.
- ROM over the gait cycle

All trials were time-normalized from 0% to 100% of the gait cycle.

The calculated outcomes were used to assess intra- and inter-operator reliability and to compare gait characteristics between the healthy control group and the post-arthrodesis scoliosis group.

### 2.4. Usability Assessment

A usability evaluation is a fundamental part of medical device and protocol development and is crucial for successful implementation in clinical practice [34]. According to the international Standardization Organization 9241–11 [35], usability is defined as the effectiveness, efficiency, and subjective satisfaction of a product when used by a specific user in a specific context.

After each acquisition session, the operators were asked to complete the System Usability Scale (SUS) [36,37] to provide a global measure of usability. Additionally, both operators and participants rated the perceived difficulty of each phase of the protocol on a 5-point Likert scale (1 = very easy, 5 = very difficult). The phases evaluated included:

- Phase 1: Preparation of the instruments and marker setup (participants were not involved).
- Phase 2: Participant preparation, including undressing, anthropometric measurements, and marker placement.
- Phase 3: Recording of walking trials using the optoelectronic system.
- Phase 4: Marker removal at the end of the session.

Each phase was timed to assess the efficiency of the procedure and identify potential bottlenecks. This detailed evaluation provides insight into the practicality and acceptability of the protocol for both healthy controls and patients after spinal arthrodesis, supporting its clinical feasibility and reproducibility in routine gait assessments.

### 2.5. Statistical Analysis

For the intra- and inter-operator repeatability validation phase, the protocol was tested on a sample of at least 10 participants and two clinical operators, with each procedure repeated five times per condition. This sample size is considered adequate to assess protocol repeatability and procedural feasibility in a clinical setting, as reported in previous methodological studies on motion analysis protocols [15,32,37]. For the comparison between the healthy control group and the post-arthrodesis scoliosis group, a convenience sample was used. The purpose of this comparison was not to provide definitive clinical inference, but rather to perform a preliminary exploratory evaluation of the protocol’s ability to detect potential differences in spinal kinematics during gait. Therefore, the sample size should be considered adequate for a pilot methodological investigation aimed at assessing the feasibility and sensitivity of the proposed protocol.

Descriptive statistics were calculated for all variables and are reported as mean ± standard deviation (SD). Data normality was assessed using the Shapiro–Wilk test for all continuous variables.

For the reliability analysis, intra- and inter-operator repeatability of spinal kinematics in the pathological group was evaluated by calculating the intraclass correlation coefficient (ICC) (two-way mixed-effects model, absolute agreement, average measurements) for ROM of the upper thoracic, lower thoracic, and lumbar segments. ICC values were interpreted according to standard criteria: values between 0.50 and 0.75 indicate moderate reliability, between 0.75 and 0.90 good reliability, and values greater than 0.90 excellent reliability [38].

The second step of the analysis focused on the comparison between the pathological and control groups. Group homogeneity was assessed by comparing baseline anthropometric characteristics between groups using the Mann–Whitney U test for continuous variables (e.g., age, height, weight, body mass index). Differences in spinal ROM between groups were assessed using the Mann–Whitney U test, due to the small sample size and the violation of normality assumptions for some variables. Given the exploratory nature of the present study and following similar gait studies in the literature [39,40], no correction for multiple comparisons was applied to the gait-related parameters. Significance was set at *p* < 0.05. In addition, to examine phase-dependent differences in spinal kinematics over the gait cycle, one-dimensional Statistical Parametric Mapping (SPM1d) was applied to time-normalized angular waveforms for each spinal segment and plane [41]. The significance level was set at α = 0.05. Critical thresholds were calculated that account for the smoothness of the waveform and the temporal correlation between adjacent time points. The SPM framework provides family-wise error control across the entire gait cycle, thereby addressing multiple comparisons inherent to continuous waveform analysis. Supra-threshold clusters exceeding the critical threshold were considered significant. This approach enables reproducible identification of timing-specific alterations in spinal motion that are not captured by discrete range-of-motion metrics.

For the usability analysis, mean values and standard deviations of perceived difficulty scores for each phase of the protocol, as well as the overall System Usability Scale (SUS) score, were calculated [37]. All statistical analyses were performed using SPSS 21 software (IBM, Armonk, NY, USA) and MATLAB (R2024a, The Mathworks, Natick, MA, USA). The level of statistical significance was set at α = 0.05 for all analyses.

## 3. Results

### 3.1. Intra and Inter-Operator Repeatability Analysis

The proposed protocol was applied to quantify three-dimensional spinal kinematics during gait, normalized over the gait cycle. Regarding data distribution, the Shapiro–Wilk test showed that spinal ROM parameters and global alignment measures (CVA and SVA) were not normally distributed (*p* < 0.05). Due to the non-normal distribution of spinal ROM and global alignment parameters, spinal ROM values were log10-transformed prior to the computation of intra- and inter-operator reliability (ICC). Descriptive statistics are reported as mean ± SD on the original scale for clarity. The results of the normality assessment and descriptive statistics are reported in Table 2.

Concerning repeatability in the pathological group, intra- and inter-operator reliability for spinal ROM is summarized in Table 3.

Overall, the protocol demonstrated good to excellent intra-operator reliability across the upper thoracic, lower thoracic, and lumbar segments, with ICC values ranging from 0.737 to 0.942 for both range of motion and alignment parameters (Table 3). The highest reliability was observed in the sagittal plane for the lumbar–pelvis segment. Slightly lower ICC values were found for the upper thoracic–lower thoracic segment in the sagittal plane, although all values remained within the acceptable reliability range.

Regarding inter-operator repeatability, ICC values ranged from 0.594 to 0.956, corresponding to moderate to excellent reliability. (Table 3). The highest inter-operator agreement was observed for the CVA range (ICC = 0.956), whereas lower values were mainly found for the lower Thoracic–lumbar tilt (ICC = 0.594). A similar pattern to the intra-operator analysis was observed, with slightly lower reliability in the sagittal plane for the lower thoracic and lumbar segments.

### 3.2. Comparison Between Pathological and Healthy Subjects

To ensure the reliability of the comparison between the two study groups, baseline anthropometric characteristics were first assessed, confirming homogeneity. No statistically significant differences were found between the pathological and control groups for age, weight, height, or BMI (all *p* > 0.05; Table 4). However, given the very small control sample, the absence of statistical significance should not be interpreted as true equivalence between groups. Participants were not matched for sex due to the limited sample size, which may introduce additional variability.

Spinal ROM, collected in the first session, was then compared across the upper thoracic–lower thoracic, lower thoracic–lumbar, and lumbar–pelvis segments in sagittal, frontal, and transverse planes. The results are summarized in Table 5, with all values reported as mean ± SD, and corresponding *p*-values. Significant reductions in ROM were observed in the pathological group compared to healthy controls:

- Upper Thoracic–lower Thoracic segment: Obliquity 2.34 ± 0.83° vs. 5.06 ± 2.76°, U = 5.00, *p* = 0.013; Rotation 4.90 ± 2.63° vs. 8.09 ± 3.17°, U = 6.00, *p* = 0.019
- Lower Thoracic–lumbar segment: Tilt 2.46 ± 0.44° vs. 3.62 ± 1.13°, U = 4.00, *p* = 0.008

No statistically significant differences were found for the remaining spinal segments and planes, including lumbar–pelvis tilt, obliquity, rotation, or global alignment parameters (CVA 2.73 ± 0.96° vs. 3.67 ± 3.22°, U = 22.50, *p* = 0.768; SVA 7.80 ± 1.59 cm vs. 7.66 ± 2.29 cm, U = 24.00, *p* = 0.953).

**Figure 2:**
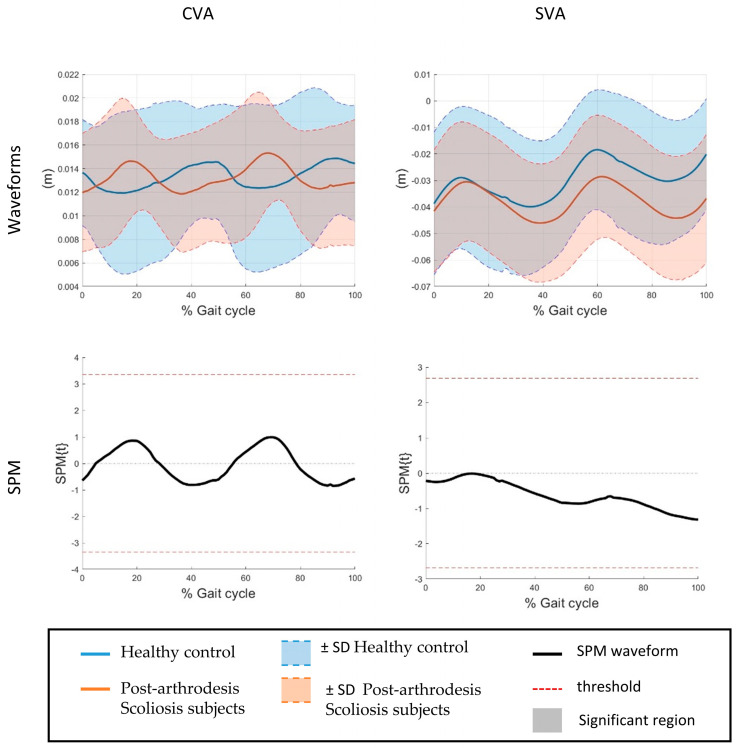
Time-normalized alignment waveforms over the gait cycle of different spine segments and relative SPM.

**Figure 3:**
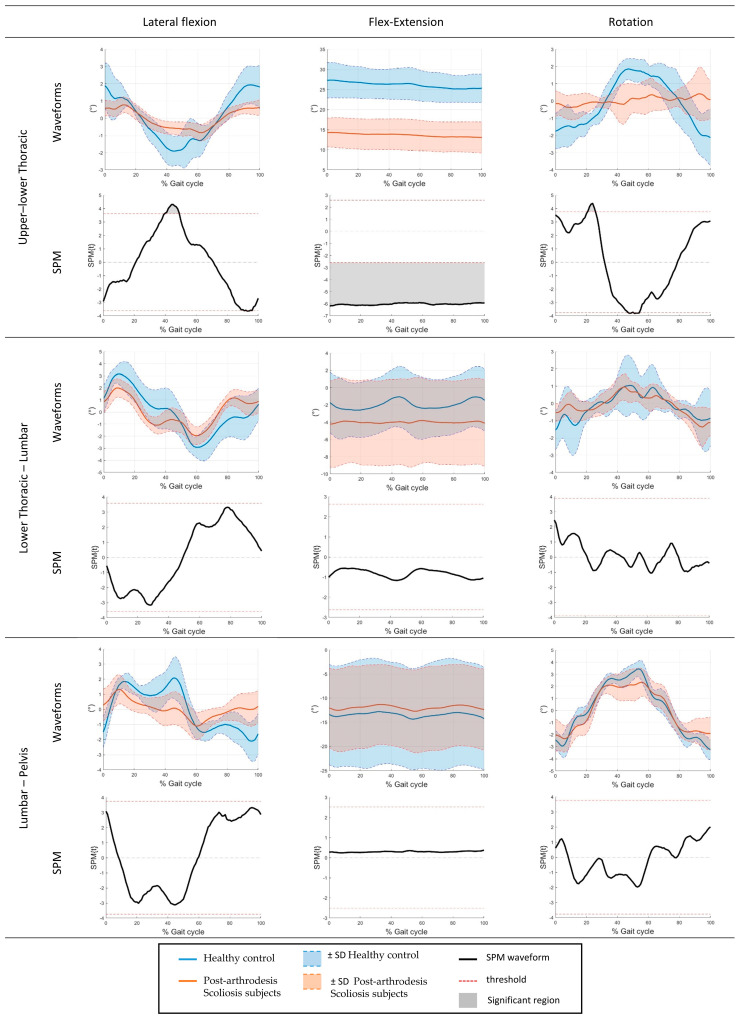
Time-normalized angular waveforms over the gait cycle of different spine segments and relative SPM.

In addition to discrete ROM measures, SPM1d was applied to the time-normalized angular waveforms over the gait cycle to identify phase-dependent differences between groups. The SPM analysis revealed significant differences exclusively at the Thoracic spinal segment. Specifically, significant supra-threshold clusters were observed in the sagittal plane throughout most of the gait cycle, in the frontal plane around mid-stance (approximately 40% of the gait cycle), and in the transverse plane during early stance (approximately 20% of the gait cycle) (*p* < 0.05). No significant supra-threshold clusters were detected for the lumbar segment or for the remaining planes of motion. The SPM results are illustrated in Figure 2 and Figure 3, which show the mean angular waveforms for both groups together with the corresponding SPM{t} trajectories and critical significance thresholds.

### 3.3. Usability Assessment

The clinical usability of the proposed protocol was assessed by collecting feedback from patients and operators during the different phases of the acquisition. Perceived difficulty scores were quantified on a 1–5 scale with higher values indicating greater ease, and, as reported in Table 6, both for patient and operator. These scores were consistently high across all phases, indicating good tolerability of the protocol even in a pathological population undergoing postoperative evaluation.

Furthermore, Table 7 reports the time required for each phase of the protocol and for the overall acquisition. Subject preparation, marker placement, and data collection were completed within clinically acceptable timeframes, without causing excessive burden to patients. Importantly, the total duration of the assessment (mean 24.04 ± 1.39 min; range 18.68–25.73 min) was comparable to that of standard gait analysis protocols and was well tolerated, also by younger participants.

The System Usability Scale (SUS), administered at the end of each session by the operator, yielded a mean score of 78.62 ± 5.06 (range 67.5–87.5), indicating good usability.

## 4. Discussion

The present study extends a previously validated marker-based protocol for spinal kinematic analysis during gait, originally tested for accuracy, repeatability, and feasibility in healthy participants, to a clinical cohort of post-arthrodesis patients. While earlier work focused on methodological validation in normative subjects, the novelty of the current investigation lies in applying the same framework to a pathological population, assessing whether the protocol maintains reliability and practical feasibility in a post-surgical context. In addition, a comparison between the pathological group and a healthy control group was conducted; however, this comparison was intended solely as a pilot and exploratory evaluation to examine the protocol’s ability to detect potential differences in spinal kinematics, rather than to draw definitive clinical inferences. By combining discrete ROM metrics with Statistical Parametric Mapping (SPM) of time-normalized waveforms, the study provides a comprehensive assessment capable of capturing both global and phase-specific alterations in spinal mobility during gait.

The protocol demonstrated good to excellent intra-operator reliability (ICC > 0.737) and moderate to good inter-operator reliability (ICC > 0.594) when applied to post-arthrodesis patients, with ICC values largely comparable to those previously reported in healthy individuals and in other optoelectronic gait analysis studies [32,42]. Reliability was highest for lumbar–pelvis kinematics, particularly in the sagittal plane, whereas slightly lower ICC values were observed for Thoracic segments, especially in sagittal motion, consistent with previous studies [32,36,42]. These results reflect known sources of variability in spinal motion capture, including soft tissue artefacts, muscle contractions, and challenges in marker placement over paravertebral and scapular regions. As commonly reported in motion capture studies, some variability can be attributed to soft tissue artefacts and marker-related issues [43]. In particular, markers on the upper thoracic region may be affected by paravertebral and scapular muscles, limiting accurate identification of bony landmarks and increasing measurement variability. Occasional partial occlusions or mislabeling of back markers during gait, especially in lateral camera views, may have contributed to lower reliability in specific planes or segments [44,45]. Importantly, despite these factors, all reliability indices remained within acceptable ranges, supporting the robustness of the protocol for use in a postoperative population.

The comparison between post-arthrodesis patients and healthy controls suggests an overall trend toward reduced spinal range of motion during gait in the pathological group. However, statistically significant differences were confined to a limited number of spinal segments and planes of motion (Table 5). Specifically, reductions were detected in upper Thoracic–lower Thoracic obliquity and rotation, as well as in lower Thoracic–lumbar tilt, while no significant differences were found for the remaining spinal segments, including the lumbar–pelvis junction. Importantly, global alignment parameters did not differ between groups (CVA *p* = 0.768 and SVA *p* = 0.953; Table 5). The preservation of global alignment aligns with previous studies reporting adaptive and compensatory mechanisms following spinal fusion, whereby non-fused segments and adjacent anatomical regions help maintain functional posture and gait stability [46,47]. In this context, the limited number of significant segmental differences observed may reflect both the subtlety of post-surgical impairments and the protocol’s sensitivity in capturing meaningful variations between groups. These findings support the ability of the proposed protocol to detect segmental differences in spinal kinematics between healthy and post-arthrodesis subjects, consistent with its intended use as a methodological and pilot evaluation tool rather than for definitive clinical inference.

The SPM analysis provided additional insight into the temporal characteristics of spinal adaptations during gait. Phase-dependent differences between post-arthrodesis patients and healthy controls were identified exclusively at the Thoracic spinal segment, confirming that alterations in spinal motion are not uniformly distributed along the gait cycle. These waveform-based findings are consistent with the discrete analysis, which showed reduced Thoracic ROM in specific planes (Figure 3), while global alignment parameters (CVA and SVA) did not differ significantly between groups (Figure 2). In the sagittal plane, Thoracic spine differences extended over most of the gait cycle, indicating a persistent reduction in flexion–extension dynamics during walking. Conversely, frontal plane differences emerged mainly around mid-stance, a phase associated with load transfer and lateral stability, while transverse plane alterations were confined to early stance, when trunk rotation contributes to step initiation and balance control. Similar phase-specific adaptations of trunk motion during gait have been reported in subjects with spinal rigidity or post-surgical constraints, suggesting that Thoracic mobility plays a key role in accommodating dynamic postural demands during walking [48]. The absence of significant supra-threshold clusters at the lumbar level, together with the lack of between-group differences in CVA and SVA, suggests that lumbar–pelvic motion and global trunk alignment may be relatively preserved or actively compensated to maintain functional gait patterns, despite localized restrictions at the Thoracic segments. This behavior is in line with previous studies reporting compensatory strategies after spinal fusion, where adjacent segments contribute to preserving overall balance and sagittal alignment during locomotion [49]. Overall, these findings reinforce the methodological value of the proposed protocol in capturing both the magnitude and timing of kinematic alterations. While the study was not designed to draw definitive clinical conclusions, the phase-specific differences detected by SPM illustrate the protocol’s sensitivity in identifying subtle segmental variations and support its use as a pilot and exploratory tool for post-arthrodesis gait assessment.

In addition to the quantitative kinematic findings, the present study confirmed the practical feasibility of the proposed protocol in a clinical setting. The overall assessment duration was less than 30 min, with individual phases, preparation, marker placement, data acquisition, and marker removal, completed within clinically acceptable timeframes. Both operators and patients reported low perceived difficulty, and the SUS yielded a score indicative of good usability (mean 78.62; SD 5.06; range 67.5–87.5). These findings—together with the consistency of the kinematic results with previous studies conducted on healthy subjects [32]—support the applicability of the protocol for routine functional evaluation of spinal mobility during gait.

From a clinical perspective, the observed reduction in Thoracic spinal mobility and its phase-dependent manifestation during gait may provide preliminary insight into functional adaptations following spinal arthrodesis. Restricted motion in the Thoracic spine could increase mechanical demands on adjacent segments or on the lower limbs, potentially contributing to compensatory strategies and long-term overuse. Although the present study was not designed to establish definitive clinical indicators, the ability of the proposed protocol to detect both localized ROM reductions and subtle timing-specific alterations suggests its potential value for exploratory assessments of spinal function during gait. Future studies with larger cohorts will be required to determine whether these kinematic measures can contribute to pre- and postoperative assessment, monitoring of recovery, or evaluation of rehabilitation outcomes in patients undergoing spinal fusion.

### Limitations and Future Perspectives

Some limitations of the present study should be acknowledged. First, the sample size was relatively small, which may have limited the detection of more differences between groups, particularly at the lumbar and lumbar–pelvic levels. Second, the pathological group was heterogeneous in terms of individual characteristics and surgical history, a factor that may have contributed to the variability observed in segmental spinal kinematics. Although this heterogeneity reflects real-world clinical conditions, it may limit the generalizability of the findings. Despite these constraints, the consistency of the observed kinematic patterns, especially the selective reductions in Thoracic spinal mobility and the preservation of global alignment parameters, in line with previous studies, supports the clinical applicability of the protocol. However, caution is warranted when extrapolating these findings to broader post-arthrodesis populations. From a methodological perspective, the combination of discrete ROM measures and waveform-based SPM analysis represents a strength of the study, but future investigations with larger samples may allow a more refined phase-specific interpretation of lumbar and pelvic adaptations during gait. Longitudinal studies would also be valuable to track changes in spinal kinematics over time after surgery and in response to targeted rehabilitation interventions.

Future studies should therefore include larger and more homogeneous cohorts, as well as longitudinal designs, to investigate how spinal kinematics evolve over time after surgery and in response to targeted rehabilitation interventions. Moreover, extending this protocol to other pathological populations, such as individuals with non-specific low back pain or degenerative spinal conditions, could further strengthen its role as a standardized, objective tool for functional spinal assessment during gait.

## 5. Conclusions

This study demonstrates that the proposed marker-based protocol provides a reliable, feasible, and clinically applicable tool for the assessment of three-dimensional spinal kinematics during gait in post-arthrodesis patients. The protocol showed good to excellent intra-operator reliability (ICC > 0.737) and high usability (mean SUS 78.62 ± 5.06), confirming its suitability for routine clinical and research applications.

In our small sample, post-arthrodesis patients exhibited selective reductions in Thoracic spinal range of motion compared with healthy controls, while global alignment measures (CVA and SVA) were preserved. These localized constraints in spinal mobility, together with phase-dependent alterations identified through Statistical Parametric Mapping—particularly during gait phases related to load acceptance, stability, and trunk rotation—are consistent with previous studies and support the ability of the proposed protocol to detect meaningful segmental differences in spinal motion between groups.

In conclusion, this protocol provides a reliable method for detecting localized and timing-specific alterations in spinal kinematics in post-arthrodesis patients. Future studies should focus on larger cohorts and longitudinal designs to track postoperative recovery and refine rehabilitation strategies, as well as extend evaluations to other pathological populations.

## Figures and Tables

**Table 1:** Mean, standard deviation (SD) and range of baseline features of subjects involved in the study.

	Baseline Features	
spinal arthrodesis group (10—1 M, 9 F)	Age [y] (mean–SD; range)	24.09–SD 14.24; 62.00–14.29
	Weight [kg] (mean–SD; range)	56.20–SD 5.81; 63.00–47.00
	Height [cm] (mean–SD; range)	161.70–SD 9.78; 181.00–150.00
	BMI (mean–SD; range)	21.62–SD 2.87; 26.22–17.40
Heathy subjects group (5— 3 M, 2 F)	Age [y] (mean–SD; range)	25.76–SD 6.82; 33.20–15.00
	Weight [kg] (mean–SD; range)	58.20–SD 7.85; 66.00–49.00
	Height [cm] (mean–SD; range)	169.40–SD 8.44; 183.00–160.00
	BMI (mean–SD; range)	21.69–SD 3.26; 25.44–17.40

**Table 2:** Results of the Shapiro–Wilk test assessing the normality of spinal ROM and alignment parameters in the pathological group (*n* = 10), considering all recorded sessions. Parameters not normally distributed (*p* < 0.05) are indicated with *.

ROM		Normal		Log 10	
		Shapiro–Wilk	*p*-Value	Shapiro–Wilk	*p*-Value
Upper Thoracic—Lower Thoracic	Tilt	0.972	0.409	0.968	0.321
	Obliquity	0.977	0.573	0.954	0.105
	Rotation	0.861	0.000 *	0.953	0.100
Lower Thoracic—Lumbar	Tilt	0.952	0.092	0.983	0.797
	Obliquity	0.959	0.153	0.973	0.433
	Rotation	0.932	0.019 *	0.989	0.952
Lumbar—Pelvis	Tilt	0.950	0.074	0.983	0.791
	Obliquity	0.923	0.010 *	0.972	0.429
	Rotation	0.969	0.331	0.971	0.379
Range CVA		0.915	0.006 *	0.978	0.612
Range SVA		0.972	0.426	0.970	0.359

**Table 3:** ICC values for intra and inter operator reliability and their levels (+++ = excellent, ++ = good, + = moderate; CVA: Coronal Vertical Axes; SVA: Sagittal Vertical Axes) [38].

Metric		Intra		Inter	
		ICC (CI 95%)	ICC Level	ICC (CI 95%)	ICC Level
Upper Thoracic—Lower Thoracic	Tilt	0.737 (0.336–0.896)	++	0.712 (0.300–0.884)	++
	Obliquity	0.924 (0.809–0.970)	+++	0.832 (0.571–0.934)	++
	Rotation	0.880 (0.677–0.954)	++	0.867 (0.642–0.949)	++
Lower Thoracic—Lumbar	Tilt	0.786 (0.452–0.915)	++	0.594 (−0.550–0.841)	+
	Obliquity	0.942 (0.855–0.977)	+++	0.892 (0.724–0.957)	++
	Rotation	0.869 (0.668–0.948)	++	0.654 (0.164–0.860)	+
Lumbar—Pelvis	Tilt	0.942 (0.857–0.977)	+++	0.859 (0.644–0.944)	++
	Obliquity	0.897 (0.737–0.959)	++	0.796 (0.498–0.918)	++
	Rotation	0.931 (0.824–0.973)	+++	0.889 (0.644–0.960))	++
Range CVA		0.933 (0.831–0.974)	+++	0.956 (0.889–0.983)	++
Range SVA		0.879 (0.691–0.952)	++	0.807 (0.468–0.926)	++

**Table 4:** Comparison of baseline characteristics between pathological and healthy groups.

	Healthy Subjects (Mean–SD)	Pathological Subjects (Mean–SD)	Mann–Whitney U	*p*-Value	Effect Size
Age	25.76–6.82	24.09–4.24	16.00	0.270	0.28
Weight	58.20–7.85	56.20–5.81	18.50	0.425	0.21
Height	169.40–8.44	161.70–0.78	13.50	0.158	0.36
BMI	21.69–3.26	21.62–0.87	18.00	0.391	0.22

**Table 5:** Comparison of spinal ROM and alignment parameters (CVA, SVA) between post-arthrodesis patients and healthy controls. Group differences were assessed using the Mann–Whitney U test. Parameters showing statistically significant differences (*p* < 0.05) are indicated with an asterisk (*).

		Healthy Subjects (Mean–SD)	Pathological Subjects (Mean–SD)	Mann–Whitney U	*p*-Value	Effect Size
Upper Thoracic—Lower Thoracic	Tilt	3.58–1.74	2.66–0.43	17.00	0.371	0.25
	Obliquity	5.06–2.76	2.34–0.83	5.00	0.013 *	0.63
	Rotation	8.09–3.17	4.90–2.63	6.00	0.019 *	0.60
Lower Thoracic—Lumbar	Tilt	3.62–1.13	2.46–0.44	4.00	0.008 *	0.67
	Obliquity	6.80–1.99	4.73–1.43	10.00	0.075	0.47
	Rotation	6.94–3.15	5.14–2.65	14.00	0.206	0.35
Lumbar—Pelvis	Tilt	3.25–0.41	2.92–0.88	16.00	0.310	0.29
	Obliquity	5.57–1.89	3.82–1.43	10.00	0.075	0.48
	Rotation	8.61–2.43	6.26–2.20	13.00	0.165	0.38
Range CVA		3.67–3.22	2.73–0.96	22.50	0.768	0.08
Range SVA		7.66–2.29	7.80–1.59	24.00	0.953	0.03

**Table 6:** Assessment of the difficulty of the application of the protocol (1: very difficult–5: very easy). Phase 1 corresponded to the instrument preparation; Phase 2 was the subject preparation; in Phase 3, recordings of the movements with the optoelectronic system were carried out; and Phase 4 was the removal of the markers from the subject. The columns with colored background correspond to the operators’s votes, instead the white background corresponds to the patients’s votes.

	Operators				Patient		
Phase	1	2	3	4	2	3	4
Mean	4.78	4.88	4.73	4.93	4.68	4.73	4.63
SD	0.42	0.33	0.45	0.27	0.47	0.60	0.49
MAX	5.00	5.00	5.00	5.00	5.00	5.00	5.00
min	4.00	4.00	4.00	4.00	4.00	2.00	4.00

**Table 7:** Time (in minutes) required for the entire acquisition and for each phase.

Time to Complete the Protocol (Also Divided into the 4 Phases)					
Phase No.	1	2	3	4	Total
Mean [min]	1.81	15.16	5.30	1.76	24.04
SD	0.15	1.30	0.36	0.11	1.39
MAX [min]	2.18	16.52	6.97	1.98	25.73
Min [min]	1.48	10.02	4.82	1.62	18.68

## Data Availability

The data presented in this study are available on request from the corresponding author.

## Abbreviations

The following abbreviations are used in this manuscript:

ROM	Range Of Motion
SPM	Statistical Parametric Mapping
ADLs	Activities of Daily Living
SD	Standard Deviation
M	Male
F	Female
SVA	Sagittal Vertical Axis
CVA	Coronal Vertical Axis
ICC	Intraclass Correlation Coefficient
SUS	System Usability Scale
